# Balancing inflammation under stress: glucocorticoid regulation of innate immune pathways

**DOI:** 10.1042/BSR20250146

**Published:** 2026-09-22

**Authors:** Kirsten M. Kenney, Sabrina S. Burgener

**Affiliations:** Institute for Molecular Bioscience, The University of Queensland, Brisbane, QLD 4072, Australia

**Keywords:** glucocorticoids, inflammation, innate immunity, stress

## Abstract

Despite our constant exposure to potential dangers, our ability to withstand infection and tissue damage relies on a network of coordinated cellular security systems. One of these security features is our innate immune system, which senses danger signals from invading pathogens as well as host-derived stress signals while remaining largely unresponsive to normal host molecules and the flora of the host microbiota. This intertwining network of cellular guardians expresses pattern recognition receptors to recognise specifically pathogen-associated molecular patterns and danger-associated molecular patterns and elicit an inflammatory response. Inflammation is an integral process to eliminate a pathogenic threat while simultaneously promoting tissue repair. However, a balance exists, and persistent or aberrant inflammation can instead drive tissue damage and disease. To douse inflammatory flames, the endocrine system releases endogenous glucocorticoids, which possess potent immunomodulatory actions. This review will explore the cross-talk between endogenous glucocorticoid signalling and innate immune signalling pathways

## Innate immune signalling in infection and disease

Innate immunity was initially conceptualised as a non-specific response to microbial pathogens. Then, in the mid-1990s, toll-like receptors (TLRs) were discovered to specifically recognise pathogen-associated molecular patterns, thereby introducing the concept of pattern recognition receptor (PRR) expression on innate immune cells, as reviewed nicely elsewhere [1–4]. PRRs were subsequently found to be expressed widely on innate immune cells, including monocytes, macrophages, dendritic cells, and neutrophils, and on non-immune cells, such as epithelial cells [2,5]. PRRs are expressed within the cytosol or cellular membranes (e.g., plasma membrane or on endosomes), facilitating a highly dynamic and coordinated response to a threat. Major families of PRRs include the retinoic acid-inducible gene 1-like receptors (RLRs), C-type lectin receptors (CLRs), NOD-like receptors (NLRs), and TLRs [6].

TLRs are generally localised to the cell surface, acting as the steadfast lighthouse for alerting other PRRs of potential threats and initiating an appropriate inflammatory response. TLR-induced responses are often mediated by three major signalling programs: NF-κB-, MAPK-, or IRF-pathways [7]. However, these programs often do not occur in isolation, and concurrent signalling enhances the complexity of mounting an appropriate response. As such, initiating these signalling programs involves a transcriptional cascade of signalling molecules. For example, TLR4 engagement with lipopolysaccharide (LPS) triggers a signalling cascade that activates the NF-κB transcription machinery, which mediates the transcription of a variety of inflammatory cytokines (e.g., pro-IL-1β, TNF, and IL-6), and other PRRs like NLR family pyrin domain-containing 3 (NLRP3) [1]. NLRP3 responds to persistent threat by launching the assembly of a cytosolic multiprotein signalling complex, the inflammasome, culminating in the production of inflammatory mediators, such as cytokines, that orchestrate the next layer of a complex response.

Such a response to danger can be triggered by a pathogenic challenge and its subsequent timely resolution following pathogen clearance. Inflammation in infection can be induced retrospectively or prospectively [8]. A prospective mode of inflammation involves the detection of infectious pathogens by PRRs, such as TLRs, CLRs, and RLRs, which triggers an inflammatory response before the pathogen has the opportunity to cause tissue damage. Alternatively, an infection causes tissue damage, and the subsequent release of danger-associated molecular patterns is detected by NLRs to evoke an inflammatory response retrospectively. Prospective inflammation is host-favourable, as it allows for pre-emptive measures, such as antibacterial defences, pathogen clearance, and minimal tissue damage. Successful inflammatory responses eliminate the threat and terminate the response. However, this alone is insufficient to cease inflammation, and the lack or dysregulation of negative feedback loops, or the magnitude of inflammatory response, can exceed the threshold of homeostatic regulation. This is the switching point at which inflammation enters chronicity and drives disease exacerbation.

When inflammatory pathways are persistently or inappropriately activated, the same mechanisms that promote pathogen clearance and homeostatic restoration can instead drive disease pathology. This pathogenic role of inflammation is increasingly recognised as a unifying feature of chronic inflammatory diseases [9]. Indeed, chronic inflammation is associated with more than 50% of all deaths worldwide, attributed to inflammation-related diseases such as cancer, stroke, ischemic heart disease, metabolic dysfunction-associated steatotic liver disease (MASLD), diabetes mellitus, obesity, chronic kidney disease, autoimmune conditions, and neurodegenerative disorders [10]. The existence of potent inflammatory pathways that exert protective or pathogenic responses raises an important question: How do our bodies’ physiological systems maintain a precise balance between effective host defence and the prevention of chronic inflammation?

## The physiology of stress

Our daily life depends on many physiological processes that act in symbiosis with each other; these physiological processes are controlled by our neuroendocrine system and, in part, through the release of endogenous glucocorticoids. Glucocorticoids are steroid hormones that are essential for the daily functions of mammals, playing a pivotal role in metabolism, cardiovascular function, immune response, tissue development, and electrolyte balance [11]. Cortisol is the predominant glucocorticoid in humans and is fundamental to the human stress response; hence, it is infamously named the ‘stress hormone’. Cortisol is released in a diurnal circadian fashion [12], with peak levels in the morning to facilitate waking, followed by a gradual decrease during the day. Cortisol levels surge to provide energy in response to stress-provoking stimuli [13]. Importantly, the perception of stressful stimuli varies between individuals [14], and while short-term exposures are adaptive, prolonged stress exerts dramatic physiological and psychological consequences [15].

**Figure 1:**
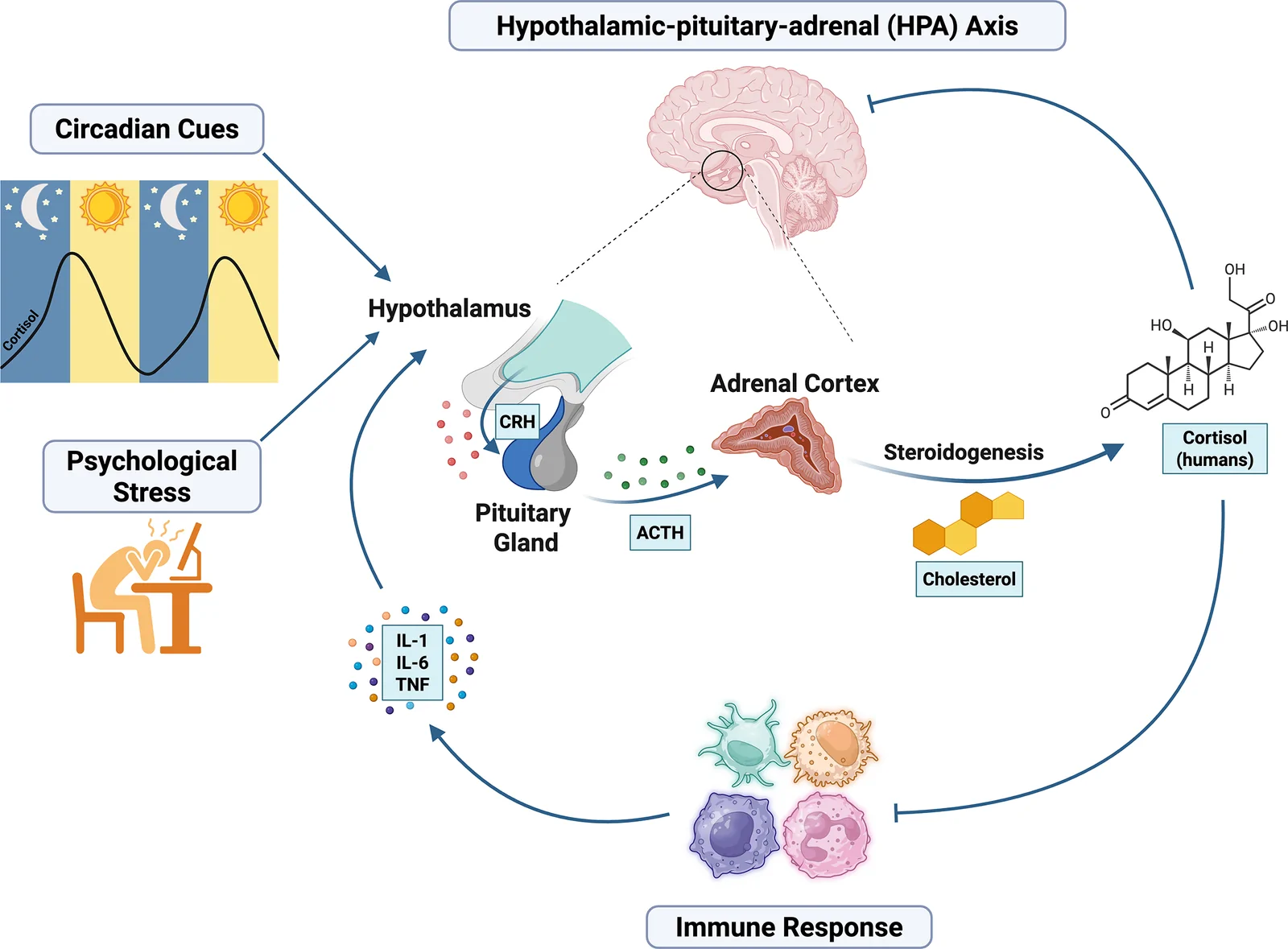
The HPA axis regulates cortisol production The hypothalamus responds to circadian cues, stress, and inflammatory cytokines (e.g., IL-1, IL-6, and TNF) by producing CRH, which acts on the pituitary gland, thereby activating the HPA axis. The pituitary gland releases ACTH, which stimulates steroidogenesis, the process by which cortisol is synthesised from cholesterol. Cortisol is released by the adrenal glands and distributed throughout the body, exerting a negative feedback loop by dampening the immune response and negatively regulating the HPA axis. Figure created using BioRender.com.

In the initial stage of an acute stress response, the ‘fight-or-flight’ response, the amygdala in the brain’s limbic system releases adrenaline into circulation to increase blood pressure, respiration, and heart rate while vasoconstricting arterioles [16]. Notably, this short-term sympathetic response is pro-inflammatory, facilitating the destruction of pathogens [14]. The amygdala also signals to the hypothalamus, which responds to psychological stress, pro-inflammatory cytokines, or circadian cues, to produce corticotrophin-releasing hormone (CRH) (Figure 1). CRH acts on the pituitary glands and activates the hypothalamic–pituitary–adrenal (HPA) axis [17]. In turn, the anterior pituitary glands release adrenocorticotrophic hormone (ACTH) into the circulation, allowing the release of cholesterol. A process called steroidogenesis synthesises cortisol from cholesterol. The adrenal cortex releases cortisol to be widely distributed throughout the body [18] with levels rising systemically approximately 15 min after exposure to a stressor and remaining elevated for several hours [14], supporting glucose mobilisation, suppressing nonvital organ systems, and decreasing inflammation by a negative feedback loop [19–21]. Cortisol additionally blocks the activation of the HPA axis, thereby reducing CRH and ACTH production.

The concept of stress being a broadly immunosuppressive response was established by Selye’s seminal studies in the mid-20th century [22]. Half a decade later, we now recognise that glucocorticoids elicit varying cellular responses in terms of specificity and magnitude of action [23,24]. As such, a comprehensive meta-analysis of 18,941 individuals across a 30-year inquiry period reported that acute time-limited stressors, such as public speaking and arithmetic, robustly change the cellular composition of circulating immune cells with increased numbers of neutrophils, monocytes, and natural killer cells [25]. These changes are consistent with the redistribution of immune cells, mediated by acute stressors, into tissue where rapid effector function may be required, such as during a local infection [26,27]. In contrast, chronic stressors, such as caregiving, living with a disability, and unemployment, are associated with widespread impairments across innate and adaptive immune systems, accompanying the most pronounced global immunosuppression [25]. Nevertheless, this picture is not complete, as more recent work highlighted that prolonged glucocorticoid exposure, induced by stress, may paradoxically promote a pro-inflammatory immune state. For example, chronic exposure to corticosterone, the murine equivalent to cortisol, increased the gene expression of pro-inflammatory markers in the hippocampus and potentiated the pro-inflammatory response of microglia [28]. Similarly, in a rat model of repeated social stress, chronic stress increased microglial activation status, altered brain immune function, and induced a pro-inflammatory milieu [29].

Evidence from human studies similarly supports this duality. Baseline salivary cortisol levels were measured in 80 workers working in gas and oil fields [30]. Workers with >10 ng/ml cortisol presented with significantly increased body weight and body mass index, alongside increased basal gene expression of *IL1B*, *IL6*, and *IL10* in peripheral blood mononuclear cells, compared with workers with ≤10 ng/ml cortisol [30]. The increase in both anti-inflammatory (IL-10) and pro-inflammatory (IL-6 and IL-1β) cytokines appears counterintuitive. This might be due to potential glucocorticoid resistance or inflammatory override in the context of chronic stress. These findings highlight that the relationship between glucocorticoid signalling and immune modulation is dynamic and multifaceted.

Accordingly, endogenous glucocorticoids act as tightly regulated, transient signals, characterised by a short half-life and rapid binding to corticosteroid-binding globulin (CBG), which confers temporal and spatial control over endogenous glucocorticoid availability. CBG, also known as transcortin, is the primary carrier of circulating cortisol, thereby regulating its availability, with less than 10% of ‘free cortisol’ in circulation. Mutations or deficiencies in CBG may reduce its binding affinity, leading to dysregulated cortisol binding. Several genetic mutations of CBG (e.g., CBG Leuven [31], CBG Lyon [32], CBG Null [33], CBG G237V [34], CBG Santiago [35], CBG Athens [36], and CBG Montevideo [37]) have been associated with clinical symptoms of fatigue, weight gain, idiopathic pain, and hypotension, which may be attributed to increased cortisol binding to albumin rather than CBG, as observed in both human CBG-deficiency [32,38] and mouse models [39]. These findings have contributed to the ‘reservoir hormone hypothesis’, proposing CBG is not a mere passive transporter of cortisol but rather a functional mobile reservoir [40]. In this capacity, CBG may facilitate the retention and controlled release of cortisol in response to physiological demand. This notion is further supported by studies suggesting CBG is necessary to mount an appropriate stress response [39,41], potentially through targeted delivery of cortisol to sites of inflammation. At sites of inflammation, activated neutrophils release high concentrations of neutrophil elastase, which cleaves CBG, disrupting the cortisol binding site, thereby reducing binding affinity and releasing cortisol locally to modulate the inflammatory response [42–45].

## Cross-talk between innate immunity and endogenous glucocorticoids

Mutual cross-talk and balance between the innate immune system and the endocrine system ensure an organism’s survival and appropriate response to stress. Following chronic stress or prolonged immune activation, this cross-talk and mutual regulation can be impaired. Historically, much of our understanding of the anti-inflammatory and immunosuppressive effects of glucocorticoids has been derived from studies utilising saturating concentrations of synthetic glucocorticoids, such as prednisolone and dexamethasone [46]. This has developed an oversimplified view of the immunomodulatory actions of glucocorticoids and may have overlooked vital distinctions between the physiological actions of endogenous glucocorticoids and, by extension, the chronic stress response.

### Glucocorticoid mechanisms of transcriptional regulation

Since glucocorticoids are lipophilic, they readily traverse the plasma membrane and engage with intracellular receptors, which function as ligand-activated transcription factors (TFs) to regulate the transcription of target genes. Cortisol binds to two receptor classes, the glucocorticoid receptor (GR) family, GRα/GRβ, and the mineralocorticoid receptor (MR) family [47]. Endogenous cortisol binds the MR with high affinity [48,49], resulting in near-maximal occupancy under basal conditions, whereas activation of the lower-affinity GR requires higher cortisol concentrations, such as those reached during circadian peaks or stress [50,51]. By contrast, synthetic glucocorticoids are engineered to preferentially bind the GR [48,52], a property closely linked to their potent anti-inflammatory efficacy [17,46,53].

The MR binds aldosterone with similarly high affinity as cortisol [54], despite cortisol circulating 100-fold the concentration of aldosterone [55], underpinning its critical role in regulating electrolyte homeostasis and blood pressure [56]. Dysregulated MR signalling has been implicated in the pathogenesis of cardiovascular disease through its role in mediating aldosterone signalling [57]. This is evidenced by the clinical success of MR antagonists, such as eplerenone and spironolactone, which have been shown to reduce cardiovascular morbidity and mortality [58–60]. The MR is largely engaged under basal physiological conditions, while the GR predominates during dynamic or stress-responsive states. Indeed, the MR can be occupied by glucocorticoids but not activated [61,62], and exposure to chronic stressors down-regulates hippocampal MR expression [63,64].

The MR and GR share substantial overlap in their expression patterns, ligand binding, and transcriptional effects [65,66], as well as 94% amino acid homology in their DNA binding domain [67], which permits heterodimerisation and functional cross-talk. Heterodimerisation between the MR and GR has been shown to expand the dynamic range of glucocorticoid-mediated transcriptional responses [68,69], further highlighting the complexity of glucocorticoid signalling. Important functional distinctions exist between the two nuclear receptors. The GR exerts greater transcriptional versatility in transrepressive pathways, including greater potency in transrepression of AP-1 [70]. In general, the GR plays a more dominant role in mediating glucocorticoid-mediated transcriptional activity during stress, particularly in regulating the transcription of inflammatory cytokines, compared with the MR.

**Figure 2:**
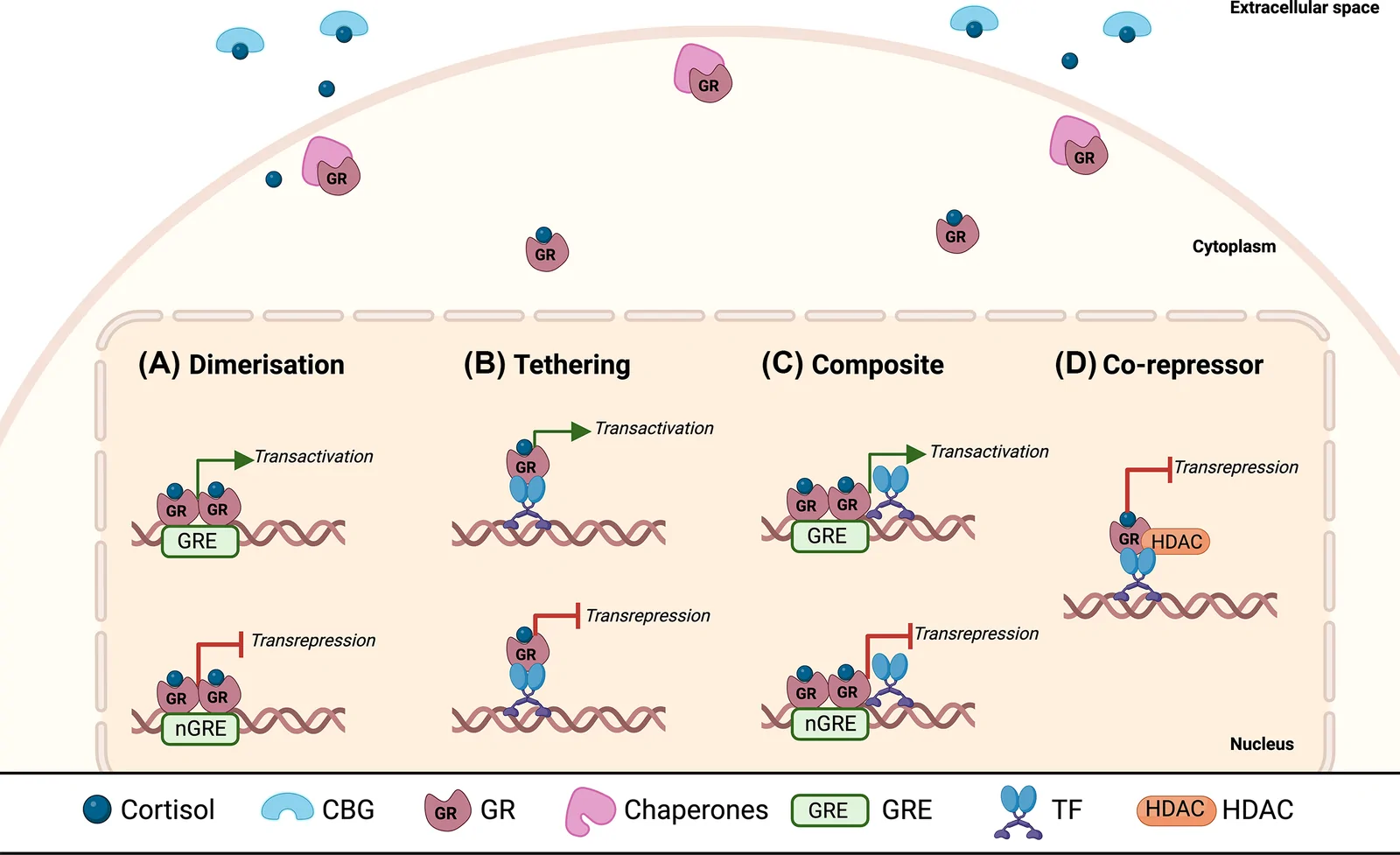
Mechanisms of transcriptional regulation by the GR Circulating cortisol is predominantly bound to CBG, then dissociates from CBG to freely diffuse across the plasma membrane and engage with the cytosolic GR. In the absence of a ligand, GR is maintained in an inactive state bound to chaperones. Upon cortisol binding, the activated GR complex translocates to the nucleus, where it regulates transcriptional activation or repression through several mechanisms: (**A**) GR dimerisation and direct binding to GRE; (**B**) GR tethering to other DNA-bound TFs without direct DNA binding; (**C**) composite regulation involving GRE binding alongside TF tethering; and (**D**) recruitment of other repressor complexes, such as HDACs. Figure created using BioRender.com.

GR engagement of endogenous glucocorticoids exerts anti-inflammatory effects through several mechanisms: (i) GR dimerisation and direct binding to glucocorticoid response elements (GRE), (ii) tethering of the GR to other DNA-bound TFs, (iii) composite of GR dimerisation alongside tethering, or (iv) recruitment of other repressor complexes, like histone deacetylases (HDACs) (Figure 2). An estimated 1000 to 2000 genes are subjected to GR-mediated regulation [11,71]. These endogenous functions have been leveraged for the design of synthetic glucocorticoids and, independently, play an important role in regulating the transcription of inflammatory pathways.

#### GR dimerisation

The GR rapidly cycles on and off GREs, presumably to allow the GR to sample multiple binding sites and interaction proteins [72,73]. The GR can regulate gene transcription through transactivation or transrepression by promoting the expression of anti-inflammatory genes or inhibiting the expression of pro-inflammatory genes, respectively. Direct binding of the GR to GREs is observed in numerous genes, including glucocorticoid-induced leucine zipper (GILZ) [74], serum and glucocorticoid-regulated kinase 1 [75], and mitogen-activated protein kinase phosphatase-1 [76]. GR dimerisation was reportedly necessary to suppress IL-1α and IL-1β in a murine septic shock model [77]. Macrophages from GR-dimerisation-deficient mice did not down-regulate IL-1β in response to synthetic glucocorticoids, and GR-dimerisation-deficient mice succumbed to IL-1β-dependent death upon LPS-induced shock *in vivo* [77]. Interestingly, most genes negatively regulated by the GR do not contain a classical GRE [78], indicating alternative regulatory mechanisms, such as GR tethering to other TFs, influence their function.

#### GR-tethering and composite binding

The GR can regulate gene expression either by physically tethering DNA-bound TFs [23,79] or by composite regulation of GRE binding, along with TF tethering. GR-tethering is regarded as a primary mechanism by which glucocorticoids mediate inflammatory dampening. Tethering of GR to specific TFs has also been shown to enhance the transcription of responsive genes. As an example, the GR tethers to the Jun subunit of AP-1 [80] and the p65 subunit of NF-κB [81,82] to suppress their transcriptional activity, acting as a transrepressor. While GR tethering to STAT5 enhances the transcription of β-casein; an essential protein in mammalian milk, thereby acting as a transactivator [83]. Whereas binding of GR to STAT3 and to neighbouring DNA sites leads to transcriptional synergism [84], highlighting a composite mechanism of transcription regulation by GR dimerisation and tethering in concert.

#### GR recruitment of repressor complexes

Glucocorticoids may also influence gene transcription through GR-mediated recruitment of other repressor complexes, such as HDACs. Given their name, HDACs deacetylate histones at the promoter region of genes of interest, thereby condensing chromatin and suppressing transcription [85]. A crucial anti-inflammatory mechanism of the GR is to recruit HDAC2 to actively transcribe inflammatory genes, which then deacetylates their histones and silences gene expression [86,87]. The interaction of glucocorticoids and the GR within the chromatin landscape has been previously explored [88–91]. However, most of these studies assessed synthetic glucocorticoids, and given endogenous and synthetic glucocorticoids differ considerably in their immunomodulatory actions, future studies investigating the impact of the former within the chromatin landscape would be highly insightful.

### Anti-inflammatory properties of endogenous glucocorticoids

The anti-inflammatory effects of pharmacological glucocorticoids down-regulating and inhibiting NF-κB signalling are well documented [92–97]. Physiologically, cortisol limits excessive inflammation by quenching pathophysiological responses to injury and inflammation, thereby preventing further tissue damage [98]. At the transcriptional level, cortisol blocks NF-κB transcriptional activity, consequently down-regulating the pro-inflammatory cytokines, IL-1, IL-6, TNF, and IFN-γ, while simultaneously inducing anti-inflammatory cytokines, TGF-β, and IL-10, and their respective receptors, TGF-βR and IL-10R [93,99]. Despite this, chronic stress, accompanied by sustained elevations in cortisol levels, is associated with enhanced NF-κB activity [100–104], suggesting a shift toward transcriptional duality rather than solely inflammatory restraint. At the level of innate immune sensing, glucocorticoids also regulate PRR expression and signalling capacity. For example, in rat peritoneal macrophages stimulated with the TLR2 agonist Pam3CSK4, corticosterone pre-treatment significantly reduced TNF production and dose-dependently down-regulated TLR2, but not TLR4, gene expression [105]. Collectively, these findings highlight glucocorticoids selectively tune PRR responsiveness, rather than uniformly suppress.

Many cell signalling programs auto-regulate through a transcriptional negative feedback loop. A central mechanism underpinning glucocorticoid-mediated effects is the induction of negative feedback regulators, such as GILZ [106]. GILZ suppresses the production of pro-inflammatory chemokines and cytokines [107] and is constitutively expressed in many cell types [108–110]. Mechanistically, GILZ acts as an NF-κB binding partner to block NF-κB-mediated pro-inflammatory gene transcription [107,111], as well as exerting TF functions independently of NF-κB [112–114]. The ability of GILZ to mediate anti-inflammatory effects resides in its influence of blocking gene transcription by homo- and heterodimerisation with other TFs, such as NF-κB and AP-1 [107,115], thereby broadening its regulatory influence. Studies suggest that GILZ is required to mediate many of the anti-inflammatory effects of glucocorticoids [107,111–116], positioning it as a central mediator of glucocorticoid-mediated transcriptional effects.

### Context-dependent immunomodulation

Endogenous glucocorticoids exert context-dependent effects on innate immune functions, with their ability to either augment or suppress the production of inflammatory mediators shaped by concentration, duration of exposure, and cell type. For example, in a murine system, low corticosterone concentrations (the murine equivalent to human cortisol) can enhance macrophage activation, whereas higher concentrations typically suppress inflammatory responses. This dose-dependent effect has been demonstrated in RAW264.7 mouse macrophage-like cells, wherein low corticosterone concentrations up-regulated LPS-induced protein expression of NLRP3, while high corticosterone concentrations down-regulated NLRP3 expression [117]. Similar findings were reported in BV2 mouse microglia-like cells, where 24-h pre-treatment with 50 nM corticosterone increased LPS-induced IL-1β expression, whereas 500 nM corticosterone attenuated IL-1β [118]. A switch in receptor preference was postulated to underpin this; low concentrations of corticosterone agonise the MR, while high concentrations agonise the GR [118]. Supporting this notion, a separate study reported that low corticosterone concentrations exerted immunostimulatory effects on macrophage function and similarly investigated the role of the GR [119]; notably, both studies used mifepristone to assess GR involvement [118,119]. However, mifepristone harbors off-target effects, as it antagonises the GR and progesterone receptor and suppresses NLRP3 activation [120,121], thereby complicating these interpretations.

Endogenous glucocorticoids exert context-dependent inflammatory effects through transcriptional regulation across multiple biological systems. In bovine endometrial epithelial cells, 12 h of cortisol exposure down-regulated the expression of *TLR4, IL1B, IL6, IL8, TNF, COX2*, and *INOS*, alongside suppressing the phosphorylation and degradation of IκBα, thereby preventing NF-κB activation [122]. Consistent with these immunomodulatory effects, elevated cortisol levels have been observed in postpartum cattle [123], a period associated with a high incidence of clinical endometriosis [124,125]. A similar association between endogenous glucocorticoids and endometriosis has also been observed in humans, where women with endometriosis reported heightened perceived stress [126], coinciding with increased cortisol [127,128]. More broadly, stress-induced increases in cortisol levels have been implicated as a compounding factor in chronic inflammatory diseases such as vitiligo [129,130]. In a monobenzone-induced vitiligo-like *in vivo* model, restraint stress exacerbated depigmentation and enhanced NLRP3 inflammasome signalling, including increased expression of caspase-1, ASC, GSDMD-N fragment, NLRP3, and IL-1β [131]. Pharmacological inhibition of NLRP3 with MCC950 rescued the vitiligo-like phenotype [131], indicating that stress-mediated glucocorticoid responses can enhance innate immune activation.

The immunological consequences of glucocorticoid signalling are highly context-dependent, differing substantially between acute, transient exposure and chronic elevations associated with prolonged stress. Notably, chronic glucocorticoid exposure is often intertwined with metabolic and circadian dysregulation, reinforcing the concept that glucocorticoid-immune cross-talk operates within a broader physiological network, rather than as a unidirectional immunosuppressive pathway.

## Stress as a co-morbidity in disease

**Figure 3:**
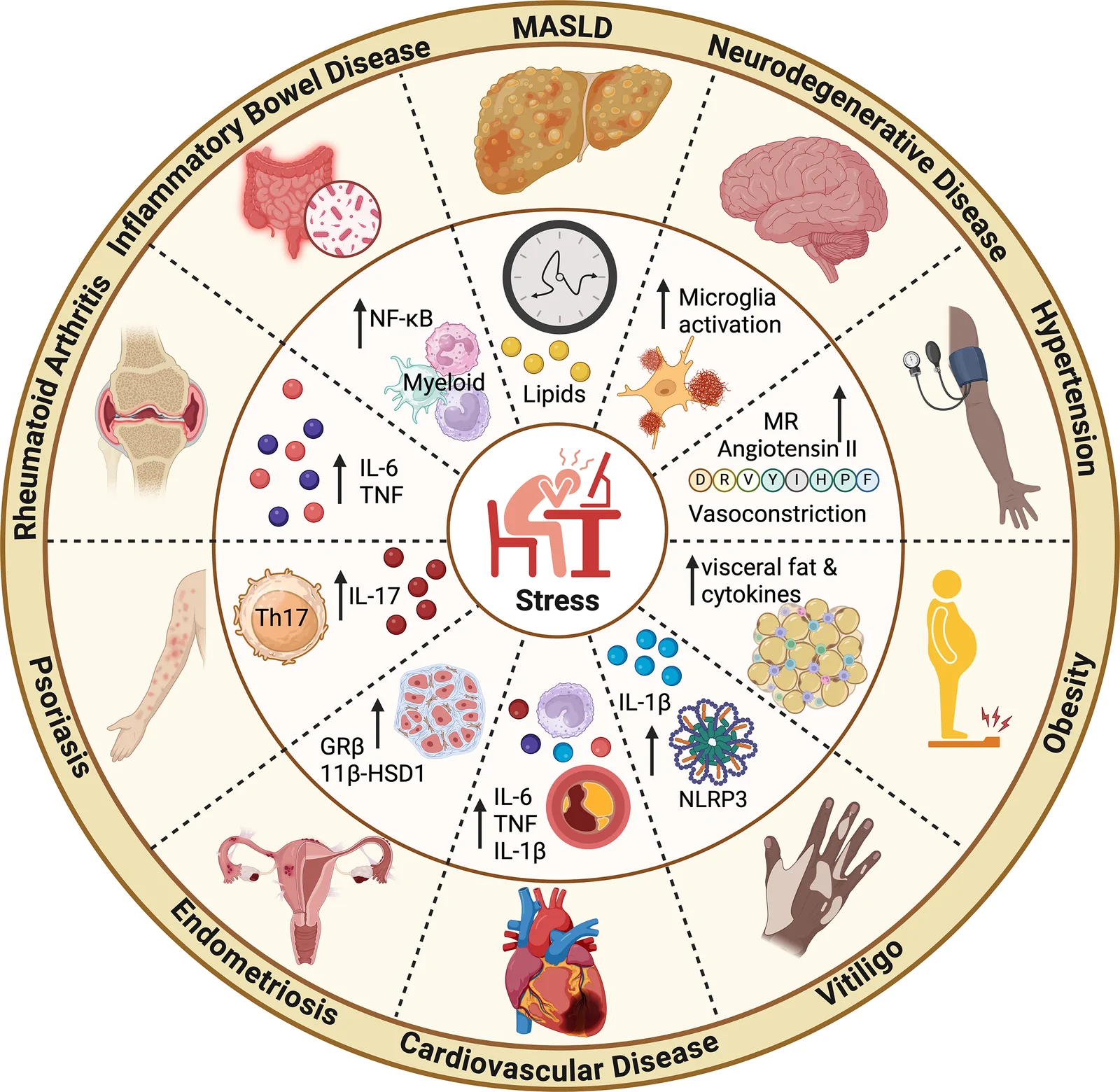
Stress is a central risk factor in disease Stress is highly associated with numerous disease states, including MASLD, neurodegenerative disease, hypertension, obesity, vitiligo, cardiovascular disease, psoriasis, endometriosis, rheumatoid arthritis, and IBD, acting pro-inflammatory via up-regulation of innate immune pathways. Figure created using BioRender.com.

Stress is a common risk factor for 75%–90% of diseases [20,132]. Disruption in the balanced interplay between the innate immune and endocrine systems can precipitate a host of pathologies (Figure 3), such as MASLD, inflammatory bowel disease (IBD), neurodegenerative disease, and rheumatoid arthritis.

### Metabolic dysfunction-associated steatotic liver disease

MASLD is an umbrella term describing a spectrum of diseases, including metabolic dysfunction-associated steatotic liver, with simple steatosis, and metabolic dysfunction-associated steatohepatitis, which can further progress to cirrhosis and hepatocellular carcinoma [133]. MASLD is strongly associated with obesity and insulin resistance, making it an emerging global healthcare problem [134], with more than one-third of the global population estimated to have MASLD [135,136]. Beyond metabolic risk factors alone, accumulating evidence implicates stress as a risk factor for MASLD development and progression [137,138], as prolonged stress heightens fatty acid synthase activity, contributing to weight gain, inflammation, and increased liver disease burden [139–142]. Consistent with this, a meta-analysis of longitudinal studies found a positive correlation between weight gain and stress [143], and stressed individuals gravitate towards calorie-dense foods [144–146], further amplifying the vicious cycle. Parallel findings in animal models show that rats exposed to various stressors favour stress-induced eating of lard and sucrose [147,148]. Moreover, increasing stress levels over time were associated with a higher incidence of MASLD in a cohort of female nurses [149], underscoring the relevance of stress as an enhancer of metabolic disease risk.

Stress also intersects with metabolic disease through disruption of circadian regulation, which plays a critical role in coordinating innate immunity and metabolic pathways. The circadian clock tightly regulates numerous inflammatory processes [150,151], and disruptions to the circadian clock are linked to obesity [152]. A prime example of chronically disrupted circadian rhythm is in night shift workers, who correlatively experience a higher rate of metabolic disorders and obesity [152]. This is further compounded by the complex and reciprocal relationship between circadian rhythm and stress, whereby chronic stress disrupts circadian rhythm, and a disrupted circadian rhythm can increase stress [153,154], creating a feed-forward loop that may potentiate metabolic inflammation.

Mechanistic insight into the interaction between stress and hepatic inflammation has been assessed in murine models. Wistar rats subjected to chronic restraint stress developed marked liver pathology, accompanied by increased hepatic levels of IL-1β and NLRP3 expression, compared with control rats; a phenotype recapitulated by administration of exogenous corticosterone [155]. Pharmacological blockade of glucocorticoid signalling using mifepristone (GR antagonist) or metyrapone (11β-hydroxylase antagonist) attenuated these pathologies [155], indicating stress-induced glucocorticoid elevations exacerbate metabolic liver injury. These findings can be interpreted as direct evidence for glucocorticoid-induced activation of the NLRP3 inflammasome; however, an alternative explanation is that glucocorticoid-driven metabolic perturbations, such as lipid accumulation and hepatic damage, secondarily activate NLRP3 inflammasome signalling [156–159]. Altogether, these studies suggest chronic exposure to stress, and by extension, heightened glucocorticoid signalling, promotes metabolic disease through coordinated effects on eating behaviour, circadian regulation, and innate immune activation.

### Inflammatory bowel disease

IBD describes a group of chronic intestinal inflammatory diseases, including ulcerative colitis and Crohn’s disease. Stress disrupts the gut microbiome and intestinal permeability [160,161], and accumulating evidence suggests chronic stress elicits the deterioration and relapse of IBD. In Wistar rats, swimming-induced stress increased gastrointestinal permeability, an effect ablated by adrenalectomy or pharmaceutical inhibition of the GR [162]. Cell-specific deletion of *Nr3c1* (GR) in enteric neurons and glia (*Nr3c1*^Hand2^ mice) protected these animals from stress-induced colitis and monocyte accumulation [163], highlighting the interaction of the brain-gut axis. Moreover, in a dextran sulfate sodium (DSS)-induced colitis model, intestinal epithelium GR knockout mice (*Nr3c1*^ΔIEC^) exhibited notably reduced disease activity and tissue damage [164]. On the other hand, chronic restraint stress exacerbated colitis progression, with increased immune cell infiltration, degradation of the colonic mucus barrier, and disrupted gut microbial composition [165]. This inflammatory exacerbation fosters dysbiosis by increasing the composition of inflammation-promoting microbes, such as *Streptococcus*, *Helicobacter*, and *Enterococcus faecalis*, in stressed mice relative to normal control mice [165]. In line with this, stress exacerbated intestinal inflammation and disease severity in DSS-induced colitis mice models [163,166] and up-regulated the expression of *Tnf, Ccl2, and Il6* [163]. Intriguingly, stress exposure prior to colitis onset induced the greatest disease exacerbation, suggesting that enhanced levels of endogenous glucocorticoids may precondition intestinal inflammation upon subsequent encounter with a triggering stimulus [163].

The concept of stress priming the innate immune response is not new. A comprehensive study in 2021 assessed the transcriptional regulation of inflammatory genes by stress in female participants enrolled in the Heart Attack Research Program with high levels of perceived stress and female mice subjected to chronic variable stressors [167]. Transcriptomic profiling revealed enriched NF-κB, IL-6, and IFN signalling in myeloid cells of both study groups, compared with their control groups, suggesting a hyperinflammatory state induced by chronic stress [167]. Stress-induced priming of the innate immune system was further showcased in healthy male volunteers subjected to the Trier Social Stress Test, wherein stress enhanced NF-κB activation and gene expression of pro-inflammatory cytokines, *IL1B* and *IL6*, compared with non-stressed controls [103]. Stress rewiring innate immune signalling pathways to a primed state might explain why, in diseases such as IBD, patients have periodic flares.

Stress is widely recognised by patients with IBD as a contributing factor to disease pathogenesis. Approximately 70% of patients with IBD reported stress as an important factor affecting disease course, and 85% of patients indicated effective stress management positively impacted disease progression [168]. Despite these strong patient-reported associations, the mechanistic effects of stress on intestinal permeability and disease progression in humans remain less definitive. Acute stress situations, such as public speaking, increase salivary cortisol and have been linked to increased gut permeability [169]. In contrast, a similar public speaking model significantly increased cortisol levels without impairing intestinal barrier function [170]. Similarly, a study subjected 19 healthy participants to tandem skydiving, which significantly boosted salivary cortisol levels but did not induce any notable changes in gastroduodenal, small intestinal, or colonic permeability [171]. Despite this, clinical evidence indicates a clear association between heightened stress and IBD disease outcomes. In a study of 110 patients with ulcerative colitis, patients with high perceived stress had a 3.6-fold increase in the odds of clinical flares [172].

Within this context, the gut–brain axis communication operates through a broader neuroendocrine framework, in which the intestinal environment and psychological stress converge on shared regulatory pathways, including glucocorticoid signalling. Dysregulated stress responses may therefore amplify immune cross-talk between the gut and brain, with implications that extend beyond intestinal pathology.

### Neurodegenerative disease—Alzheimer's disease

Alzheimer's disease is a chronic neurodegenerative disorder primarily characterised by memory loss and cognitive decline [173]. This progressive cognitive impairment can severely interfere with daily activities. Alzheimer's disease is the most prevalent form of dementia, comprising approximately two-thirds of dementia cases in individuals aged 65 years and older [174]. The pathological hallmarks of this neurodegenerative disease are neurofibrillary tangles formed by hyperphosphorylated tau protein within neurons and the accumulation of β-amyloid extracellular plaques [175,176]. While tau tangles and amyloid plaques are hallmarks of Alzheimer's disease, contrasting evidence suggests they may not be the primary causative agents but rather collateral features of neuroinflammation, metabolic dysfunction, and aberrant immune signalling [177–180]. Especially in light of many treatments targeting amyloid and tau failing to show significant effect in clinical trials [181–184], alternative disease-driving mechanisms have gained momentum, one of them being neuroinflammation.

A systematic review and meta-analysis on 17,245 individuals found that morning cortisol levels are further elevated in Alzheimer's patients compared with age-matched cognitively healthy individuals [185]. These exacerbated cortisol levels at wakening are associated with worse memory performance in amyloid-positive patients [186], and consistently high cortisol levels in older adults are associated with impaired hippocampus-dependent memory function and hippocampal atrophy [187].

Longitudinal study designs are indispensable for identifying how molecular, immune, and cognitive changes progress across disease stages. The cognitive reserve index (CRI) defines cognitively enriching and stimulating life experiences (e.g., education, social health, occupation, physical activities, and leisure activities) and is directly associated with greater cognition [188]. However, adjusting the CRI to individually measured cortisol levels reduced this beneficial association, highlighting the negative impact stress has on reducing the neurocognitive benefits accrued over one's lifetime [188]. The Baltimore Longitudinal Study of Aging (BLSA) measured the association between cortisol dysregulation and Alzheimer's disease risk by assessing the total amount of cortisol excreted in urine (UFC) within 24 h, normalised to creatinine (Cr) levels, over 10.5 years [189]. The BLSA study found that consistent increases in UFC/Cr levels over time, or fluctuating levels, were significant predictors of Alzheimer's disease risk ∼2.9 years before disease onset [189]. Thus, posing the question: could cortisol levels be used as a diagnostic or prognostic tool for Alzheimer's risk? And by extension, are tailored interventions for stress reduction a viable option to mitigate this risk factor?

Therapies targeting glucocorticoid signalling have been implemented within the context of Alzheimer's disease, to varying degrees of success. Treatment with the GR antagonist, mifepristone (RU486), reportedly decreased Alzheimer's disease pathogenesis in mouse models [190,191], and moderately improved cognitive scores in small clinical trials [192,193]. However, there are several caveats to this therapeutic intervention. Firstly, the GR is not uniformly distributed in the central nervous system. GR immunoreactivity is high within the hypothalamus but weak within the cornu ammonis 3 region of the hippocampus [194,195], which is essential for memory encoding and spatial mapping. Thus, dysregulated glucocorticoid signalling can result in cell-type and region-specific differences. Secondly, mifepristone has been shown to harbor off-target effects on the progesterone receptor and NLPR3 activation [120,121], which is an important consideration given NLRP3 inflammasome signalling has been implicated in Alzheimer's disease pathogenesis [196–201].

Further research deciphering the complex relationship between cortisol, neurodegeneration, and its impact on cognition is needed, as well as whether the employment of stress-management strategies would be beneficial for patient outcomes. According to the World Health Organisation, the global population of people aged 60 years and older will double by 2050, and the number of people aged 80 years and older is expected to triple between 2020 and 2050. The rise in the ageing population and the overall increase in our stressful lifestyle raise cause for concern.

### Rheumatoid arthritis

Rheumatoid arthritis is a chronic immune-mediated condition characterised by persistent joint inflammation and joint deterioration [202]. Due to the destruction of bone and cartilage in joints, rheumatoid arthritis leads to loss of function and chronic pain, culminating in reduced quality of life [203]. The pathogenesis of rheumatoid arthritis is multifactorial, involving genetic predisposition, environmental factors, and autoantibodies, such as rheumatoid factor [204]. A growing body of work also implicates stress as a contributing factor to inflammatory exacerbation in this immune disease. Indeed, patients with rheumatoid arthritis have higher plasma cortisol levels compared with healthy controls, and in rheumatoid arthritis patients, increasing cortisol concentration correlates with increasing disease activity [205]. Similarly, a recent longitudinal study evaluating perceived stress and disease outcomes in a cohort of adults with rheumatoid arthritis reported elevated stress levels were significantly associated with deteriorating disease state, enhanced pain, greater fatigue, and reduced physical function [206]. Given the association between stress and increased disease activity in rheumatoid arthritis patients, it presents the question of whether stress elevates the risk of disease development.

Mixed findings have been reported by retrospective studies assessing stressful life events and rheumatoid arthritis onset. A retrospective study of 116 participants registered with the Norfolk Arthritis Register observed no difference between the frequency or occurrence of stressful life events between participants with, or without, rheumatoid arthritis [207]. In contrast, a strong association between stressful life events and rheumatoid arthritis onset occurring within one year was observed [208]. Other studies have assessed the impact of stress in inflammatory arthritis or in newly diagnosed patients. In a prospective cohort study, participants with higher perceived stress were at increased risk of developing incident inflammatory arthritis [209]. A longitudinal study of young adults newly diagnosed with rheumatoid arthritis categorised patients based on disease stage and assessed several social, physical, and clinical measures [210]. The highest level of perceived stress was observed in patients with the most severe disease, characterised by peripheral bone erosion [210]. Altogether, the emerging picture is that stress adds fuel to the inflammatory fire in a myriad of diseases.

## Therapeutic and translational perspectives

### Limitations of glucocorticoid-based therapies

Synthetic glucocorticoids have remained a cornerstone therapeutic for treating inflammation. In cases of acute inflammation, glucocorticoids are administered intravenously at high doses, whereas in chronic inflammatory cases, they are delivered orally at low doses [211]. Like most pharmaceutical interventions, there are drawbacks to glucocorticoid therapy that limit their long-term use. For example, osteoporosis [212,213], weight gain [214,215], hypertension [216,217], and impaired wound healing [218,219] are common side-effects of long-term glucocorticoid use. One of the most severe consequences of prolonged glucocorticoid treatment is an increased infection risk, which increases with dose and duration [220]. Chronic glucocorticoid excess may also precipitate Cushing's syndrome. Cushing's syndrome can arise from an excess of exogenous synthetic glucocorticoids or endogenous cortisol [221]. In the setting of hypercortisolism, Cushing's syndrome may develop through ACTH-dependent or -independent mechanisms. The former mechanism accounts for ∼80% of cases [221] and is characterised by excessive ACTH production, typically originating from pituitary tumours or, less commonly, ectopic tumours [222,223]. In contrast, ACTH-independent Cushing's syndrome results from autonomous overproduction of cortisol by dysfunctional adrenal glands [221–223]. Elevated cortisol levels suppress CRH and ACTH production through negative feedback along the HPA axis, culminating in adrenal atrophy [224]. Similarly, chronic exposure to synthetic glucocorticoids disrupts HPA axis regulation, suppressing endogenous ACTH production, leading to adrenal atrophy, and subsequent dependence on exogenous glucocorticoid replacement [224]. Despite the immunosuppressive properties of glucocorticoids, people with Cushing's syndrome exhibit increased visceral adiposity, muscle wastage, insulin resistance, and low-grade systemic inflammation [225–227].

### Glucocorticoid resistance

Glucocorticoid resistance represents a critical and often underappreciated mechanism underlying persistent inflammation despite adequate or even elevated circulating cortisol and is commonly seen with chronic administration of glucocorticoid-based therapies. Distinguishing primary glucocorticoid resistance driven by *NR3C1* mutations (GR) that directly impair GR ligand binding or nuclear translocation, from acquired resistance seen in chronic inflammatory states, is important [228]. In the latter, GR signalling becomes locally attenuated without a genetic mutation. During chronic inflammation, changes in the tissue environment, such as increased expression of the dominant-negative GRβ receptor or reduced GR affinity, may render target tissues less responsive to cortisol even when systemic hormone levels remain normal [229–231]. This is seen in diseases such as asthma, cancer, cardiovascular disease [232], and IBD, where up to a third of patients with Crohn's disease become steroid-dependent or steroid-resistant [233]. Collectively, these findings support a mechanistic distinction relevant to chronic stress biology: enhanced inflammation may reflect either direct pro-inflammatory glucocorticoid signalling or, more commonly, acquired receptor resistance that uncouples elevated cortisol from the effective suppression of innate inflammatory pathways.

### Synthetic glucocorticoids in the clinic

Synthetic glucocorticoids have long served as the weapon of choice in the therapeutic arsenal for numerous inflammatory conditions. Their first clinical use was reported in 1948 for the treatment of rheumatoid arthritis, and they remain a mainstay therapy to this day. Despite the overuse and misuse of synthetic glucocorticoids giving rise to a myriad of side-effects, it is important to recognise the substantial benefits associated with their appropriate use. In the treatment of rheumatoid arthritis, glucocorticoid therapy is recommended by the European Alliance of Associations for Rheumatology and the American College of Rheumatology as an adjunct to disease-modifying anti-rheumatic drugs [234,235]. Current guidelines emphasise short-term usage with the lowest effective dose and prompt tapering as soon as clinically feasible [234,235]. A similar treatment paradigm exists for IBD, where oral glucocorticoids are highly effective at dampening flare-ups and inducing disease remission [236]. Though once again, minimising glucocorticoid duration and efficient tapering remain hallmark recommendations of best clinical practice to limit glucocorticoid-associated consequences [237].

While synthetic glucocorticoids remain indispensable in clinical practice, their inability to engage the physiological regulatory mechanisms governing endogenous glucocorticoid signalling underscores fundamental differences between pharmacological and endogenous glucocorticoid pathways. Therefore, an improved understanding of how endogenous glucocorticoids dynamically shape innate immune pathways has important implications for clinical practice beyond conventional immunosuppression. In this context, a qualitative mathematical model has recently been developed, which integrates response, stress level, and infection dynamics to predict increasing infection periods upon increasing stress levels [238]. Further refinement and validation of such integrative models will provide valuable insight into how varying patterns of stress exposure and endogenous glucocorticoid signalling confer either protective or pathological immune outcomes.

### Sex differences

As scientists aim to better understand the workings of the human body, they routinely turn to the mouse as a representative model. Historically, chronic disease animal models have predominantly favoured male mice, although, in certain contexts, it is advantageous to study the disease state in a single sex. For example, male mice are significantly more susceptible to diet-induced obesity and MASLD compared with females, with females largely protected by estrogen [239–241]. Similarly, in cardiovascular disease and myocardial infarction *in vivo* models, female mice are typically protected from developing severe disease states [242–244]; consequently, researchers tend to favour male mice for studying disease progression. The inverse is true for *in vivo* models of autoimmune disease, such as systemic lupus erythematosus [245–247], or infection models [248,249], wherein female mice are significantly more susceptible and, as such, are favoured in exploring the disease state. Nevertheless, research paradigms have often led to a poorer understanding of how female hormones interact with disease pathogenesis. Though maybe not intentionally, disregarding the nuanced differences between sex, the historic imbalanced sex representation in preclinical studies has been reflected in human clinical trials, harbouring substantial implications for therapeutic success [250].

Following an acute forced-swim stressor, adolescent female rats displayed impaired nuclear GR translocation, an effect not observed in their male counterparts [251]. Stress induced by inescapable tail shock enhanced neuroinflammatory and behavioural responses in both male and female rats, albeit likely through distinct, sex-specific mechanisms [252]. In female rats, stress heightened serum IL-1β, while males displayed heightened *Il1b* locally in the microglia [252], suggesting chronic stress sensitizes the immune system in a comparable, but not identical, manner between males and females. Given males and females perceive stress differently and exhibit different responses to stressful stimuli, designing experimental paradigms that appropriately capture these sex-based differences is advantageous. A recent study employed a female-focused protocol of chronic social stress, alongside a male-focused protocol of chronic social defeat stress [253]. This model captures the consequences of psychological stress in a sex-appropriate manner.

The human response to a stressful stimulus has long been characterised as ‘fight-or-flight’ [254]. However, prior to 1995, females accounted for only ∼17% of subjects in studies of neuroendocrine and physiological responses to stress [255]. A sex-dependent response to stressful stimulus has been proposed, in that males are more likely to ‘fight-or-flight’, and females are better characterised as ‘tend-and-befriend’ [255]. This behavioural preference is suggested to have evolved by virtue of differential parental investment and efforts to maximise survival of self and offspring [255]. Due to the complex nature and cross-talk between the stress response and innate immune signalling, it begs the question of whether sex hormones may add another layer of complexity to this already intricate relationship.

## The cortisol enigma: unresolved pathways and future perspectives

A number of challenges are embedded within the exploration of glucocorticoid biology.

1. Tools to study endogenous glucocorticoid biology *in vivo*.
  Both the GR and MR are essential for life; global knockout of these receptors in mice is lethal at birth (for GR) [256] and soon after birth (for MR) [257]. This adds complexity to evaluating receptor contributions, especially when inhibitors, such as mifepristone for GR inhibition, exhibit off-target effects. This challenge has been somewhat overcome in models with targeted deletion of the GR in T cells [258,259], neurons [260], dendritic cells [261], and macrophages [262,263]. A recently developed murine model involving postnatal deletion of the GR studied neurogenic heterotopic ossifications post-muscle injury [264]. Both endogenous glucocorticoids and dexamethasone injection increased heterotopic ossification incidence; however, this effect was completely ablated in postnatal GR-deleted animals [264]. The present study highlights the often-overlooked contribution of endogenous stress hormones and synthetic glucocorticoids to disease exacerbation. Altogether, these data highlight the importance of advanced tools to understand both the overlap and nuanced differences between endogenous cortisol and synthetic glucocorticoid biology.
2. Model comparison.
  Another challenge associated with investigating the biology of endogenous glucocorticoids is the limitations in comparing findings between animal and human studies. In animal studies, the induction of acute stress usually lasts 2 h, while chronic stress models may last from days to weeks. In contrast, in human studies, acute stress is typically induced for 10 to 20 min, and chronic stress can only be observed under naturally longitudinal stressful circumstances, such as caregiving roles, lengthy examination periods, and stressful roles of employment, which makes the experimental design of such studies inherently challenging.
3. Direct comparison of endogenous and synthetic glucocorticoid biology.
  Identifying the Venn diagram of transcriptional overlap and distinctions between endogenous and synthetic glucocorticoids will greatly aid the delineation of their inflammatory properties. Employing the direct comparison of endogenously produced, such as cortisol, and synthetically engineered, such as dexamethasone, in both *in vivo* and *in vitro* settings, will refine our understanding of glucocorticoid biology and define the inflammatory duality glucocorticoids play in acting as both an initiator and a terminator of immune responses depending on exposure time, dose, and cellular system.
4. Synthetic glucocorticoids in macrophage immunometabolism.
  The current fragmentation in our understanding of glucocorticoid biology is highlighted by emerging research, in which glucocorticoids exert anti-inflammatory effects by initiating metabolic reprogramming in macrophages, rather than through direct transcriptional repression [265–268]. Synthetic glucocorticoids, such as dexamethasone, destabilise HIF-1α, thereby suppressing the expression of the glucose transporter GLUT1, curtailing the aerobic glycolysis that normally fuels the pro-inflammatory and antimicrobial function of LPS-stimulated macrophages [266]. Complementing this, dexamethasone engages the GR, accelerating TCA-cycle flux by engaging the pyruvate dehydrogenase complex, driving sustained production of the anti-inflammatory metabolite itaconate [265]. Similarly, transcriptomic and metabolomic profiling confirms that glucocorticoids simultaneously repress glycolysis and promote the TCA cycle [269]. GR-driven metabolic reprogramming further extends to the suppression of numerous inflammatory pathways, including TLR signalling [270], the TNF axis [271,272], p38 MAPK signalling [262], and NLRP3 inflammasome activation [273]. Collectively, these findings indicate that glycolytic suppression, TCA cycle/itaconate signalling, and temporally regulated inflammatory pathway control constitute a metabolic axis of synthetic glucocorticoid action in macrophages, offering multiple loci at which the biology of endogenous and synthetic glucocorticoids may differ.
5. Glucocorticoid nomenclature.
  Synthetic glucocorticoids and endogenous glucocorticoids differ substantially in their immunomodulatory effects. As such, it is not appropriate for studies to claim their research explores effects of the stress response while only employing synthetic glucocorticoids, especially dexamethasone, which is often mis-referred to and set equal with endogenous glucocorticoids, despite its significantly greater potency in exerting GR-mediated anti-inflammatory effects. Confounding effects of synthetic glucocorticoids, such as treatment regimens with dexamethasone or prednisolone, do not equal a physiological stress response, nor do they represent how endogenous stress hormones act. Therefore, it is important to accurately articulate the research hypothesis based on the applied model, rather than on simplified terminology of stress.

As our understanding of glucocorticoid biology continues to evolve, so too must prevailing perspectives on their role in immune regulation. The human body is equipped with numerous tightly coordinated systems to balance inflammation under stress, allowing homeostasis to be preserved. However, when these regulatory thresholds are exceeded, both stress and inflammation can transition from adaptive and transient states to chronic and pathological conditions, and this needs to be considered in upcoming clinical trials of emerging anti-inflammatory therapies.

## Open Access

Open access for this article was enabled by the participation of the University of Queensland in an all-inclusive Read & Publish agreement with Portland Press and the Biochemical Society under an agreement with CAUL.

## Abbreviations

ACTH: adrenocorticotrophic hormone
BLSA: Baltimore Longitudinal Study of Aging
CLRs: C-type lectin receptors
Cr: creatinine
CRI: cognitive reserve index
CRH: corticotropin-releasing hormone
DSS: dextran sulfate sodium
GILZ: glucocorticoid-induced leucine zipper
GR: glucocorticoid receptor
GRE: glucocorticoid response elements
HDACs: histone deacetylases
HPA: hypothalamic–pituitary–adrenal
LPS: lipopolysaccharide
MASLD: metabolic dysfunction-associated steatotic liver disease
MR: mineralocorticoid receptor
NLRP3: NLR family pyrin domain-containing 3
PRRs: pattern recognition receptors
RLRs: retinoic acid-inducible gene 1-like receptors
TLRs: toll-like receptors
TFs: transcription factors
UFC: cortisol excreted in urine
